# *Ell3* Enhances Differentiation of Mouse Embryonic Stem Cells by Regulating Epithelial-Mesenchymal Transition and Apoptosis

**DOI:** 10.1371/journal.pone.0040293

**Published:** 2012-06-29

**Authors:** Hee-Jin Ahn, Young Cha, Seok-Ho Moon, Jee-Eun Jung, Kyung-Soon Park

**Affiliations:** Department of Biomedical Science, College of Life Science, CHA University, Seoul, Korea; Michigan State University, United States of America

## Abstract

*Ell3* is a testis-specific RNA polymerase II elongation factor whose cellular function is not clear. The present study shows that *Ell3* is activated during the differentiation of mouse embryonic stem cells (mESCs). Furthermore, *Ell3* plays a critical role in stimulating lineage differentiation of mESCs by promoting epithelial-mesenchymal transition (EMT) and suppressing apoptosis. Mouse ESCs engineered to stably express *Ell3* were rapidly differentiated compared with control cells either under spontaneous differentiation or neural lineage-specific differentiation conditions. Gene expression profile and quantitative RT-PCR analysis showed that the expression of EMT markers, such as Zeb1 and Zeb2, two major genes that regulate EMT, was upregulated in *Ell3*-overexpressing mESCs. Remarkably, knockdown of Zeb1 attenuated the enhanced differentiation capacity of *Ell3*-overexpressing mESCs, which indicates that *Ell3* plays a role in the induction of mESC differentiation by inducing EMT. In contrast to *Ell3*-overexpressing mESCs, *Ell3*-knock down mESCs could not differentiate under differentiation conditions and, instead, underwent caspase-dependent apoptosis. In addition, apoptosis of differentiating *Ell3*-knock out mESCs was associated with enhanced expression of p53. The present results suggest that *Ell3* promotes the differentiation of mESCs by activating the expression of EMT-related genes and by suppressing p53 expression.

## Introduction

Pluripotency refers to the capacity of embryonic stem cells (ESCs) to differentiate into all cell types [1], [2]. ESCs possess self-renewal capacity, which is the ability to proliferate for prolonged periods while maintaining the undifferentiated state. Recently, a core set of transcription factors, including *Oct4*, *Sox2*, and *Nanog* were found to upregulate the expression of genes that control self-renewal while repressing genes that drive differentiation [3]–[6]. How ESCs overcome the constraints of their self-renewal machinery and initiate differentiation is of great interest because understanding the mechanisms underlying differentiation will facilitate the therapeutic application of ESCs in promoting lineage-specific differentiation. The findings of recent studies have led to major advances in the molecular and biochemical understanding of the transition of ESCs from the self-renewal state to early differentiation. A recent report showed that the transcriptional repressor *Rest*, which is abundantly expressed in ESCs and is a target of the *Oct3/4-Sox2-Nanog* regulatory network, is not required for the maintenance of ES cell pluripotency, but promotes cell differentiation by suppressing self-renewal genes [7].

Several signaling networks including the leukemia inhibitory factor (LIF)/Stat3, Bmp/Smad, Ras/MAPK and Calcineurin-NFAT pathways also regulate the molecular switch between ESC self-renewal and differentiation [8]–[12]. For example, Zap70 functions to modulate the balance between LIF/Stat3 and Ras/MAPK pathways to maintain the pluripotent differentiation capacity of mouse ESCs (mESCs) [13], [14].

In addition to transcription factors and signaling pathways, epigenetic processes such as DNA methylation and chromatin remodeling are essential for determining cell fate between self-renewal and differentiation [15]. However, while recent studies on the mechanisms underlying the maintenance of the self-renewing pluripotent state have improved our understanding of ESCs, how ESCs initially enter into lineage commitment is still only partially understood.

Epithelial cells form coherent tissue layers because their lateral membranes are closely attached by intercellular adhesion complexes such as tight junctions, adherens junctions, and gap junctions, whereas mesenchymal cells can move as individual cells throughout the extracellular matrix because they are nonpolarized and lack intercellular junctions [16]. Epithelial-mesenchymal transition (EMT) is the phenotypic transformation of epithelial cells into mesenchymal cells and is related to various biological changes in development and disease. Recently, it was described that calcineurin-NFAT signaling promotes EMT during the switch of ESCs from an undifferentiated state to lineage differentiation [9]. Furthermore, several ESC-specific transcription factors were shown to bind promoters of EMT-related genes [17]. Therefore, EMT appears to be an early and essential step in lineage specification of ESCs.

Ell is a 621-amino acid protein that functions as a transcription elongation factor by suppressing the transient pausing of RNA polymerase II at multiple sites on DNA from both promoter-dependent and promoter-independent templates [18]. *Ell3* is a testis-specific RNA polymerase II elongation factor, which increases the catalytic rate of transcription elongation [19]. The C-terminal domain of *Ell3* shares strong similarities to that of *Ell*, which acts as a negative regulator of p53 and regulates cell proliferation and survival [20], [21].

Here, we analyzed the role of *Ell3* in the differentiation of mESCs. We show that *Ell3-*overexpressing mESCs rapidly differentiated compared with control cells. Furthermore, *Ell3*-knock-down mESCs underwent apoptosis under differentiation conditions. We also demonstrate that *Ell3* activates EMT-inducing genes, including Zeb1, and regulates the expression level of p53. Collectively, our results identify a unique function for *Ell3* during the initiation of mESC differentiation, and we suggest that *Ell3* promotes the differentiation of mESCs by inducing EMT and suppressing p53.

## Materials and Methods

### Reagents and cell culture

The mESC line J1 (cat. # SCRC-1010) was purchased from ATCC (Manassas, VA, http://www.atcc.org). MESCs were maintained on 0.1% gelatin-coated dishes in Dulbecco's modified Eagle's medium (DMEM, Gibco Invitrogen, Carlsbad, CA, http://www.invitrogen.com) supplemented with 10% horse serum (Gibco Invitrogen), 2 mM glutamine, 100 U/mL penicillin, 100 µg/mL streptomycin (Gibco Invitrogen), 1× non-essential amino acids (Life Technologies), 0.1 mM 2-mercaptoethanol (Sigma-Aldrich, St Louis, MI, http://www.sigmaaldrich.com), and 1,000 U/mL LIF (Chemicon, Temecula, CA, http://www.chemicon.com). To form embryonic bodies (EBs), mESC colonies were trypsinized to achieve a single-cell suspension and subsequently cultured on uncoated Petri dishes in ESC medium without LIF. To induce spontaneous differentiation, mESCs were cultured in LIF-deficient ESC medium (as described above) with 500 nM all-trans retinoic acid (RA).

### Genetic modification of mESCs

*Ell3*-overexpressing (OE) mES cell lines were generated by chromosomal integration of an *Ell3* expression plasmid, which was constructed by cloning PCR-amplified *Ell3* cDNA into modified pcDNA3.1 vectors (Invitrogen, Carlsbad, CA) in which the CMV promoter was replaced with an EF1α promoter. ShRNA plasmids targeting mouse *Ell3* were purchased (RMM3981-98494969, Open Biosystems, Huntsville, AL) and used to generate a stable *Ell3-*knock-down (KD) cell line. Three independent mES cell lines were established for *Ell3-*OE and *Ell3-*KD mESCs, respectively, and all experiments were repeated in each cell line to confirm the results. Nonspecific control siRNAs were purchased from Bioneer (Daejoen, Korea), and siRNAs targeting *Ell3* were purchased from Dharmacon (Denver, CO). mESCs were transfected with either siRNA or plasmids using Lipofectamine 2000 (Invitrogen) according to the manufacturer's instructions.

### Neural differentiation of mESCs

For monoculture neural differentiation, undifferentiated ESCs were dissociated and plated onto 0.1% gelatin-coated tissue culture plates in ESC media. After 24 h, media was exchanged with neural differentiation medium prepared as a 1∶1 mixture of DMEM/F12 (Gibco) supplemented with modified N2 (25 μg/mL insulin, 100 μg/mL apotransferrin, 6 ng/mL progesterone, 16 μg/mL putrescine, 30 nM sodium selenite, and 50 μg/mL bovine serum albumin fraction V) (Gibco) and neurobasal medium supplemented with B27 (both from Gibco). Medium was replaced every 2 days.

### RNA extraction and real-time RT-PCR

Total RNA was prepared from mESCs using TRIzol (Invitrogen) and 2–5 μg of total RNA was reverse-transcribed into cDNA using the SuperScriptII^TM^ First-Strand Synthesis System (Invitrogen) according to the manufacturer's instructions. Real-time PCR was performed in triplicate with the Quantitect SYBR Green PCR kit (Qiagen, Valencia, CA, http://www.qiagen.com) and CFX96 Real-time System (Bio-Rad Laboratories, Richmond, CA http://www.bio-rad.com). For quantification, target gene expression was normalized to the glyceraldehyde 3-phosphate dehydrogenase (GAPDH) gene. The PCR primers used in this study are listed in Table S1.

### Immunoblotting

For protein analysis, cells were washed twice with cold phosphate buffered saline (PBS) and lysed with tissue lysis buffer (20 mM Tris-base, pH 7.4, 137 mM NaCl, 2 mM EDTA, 1% Triton X-100, 25 mM b-glycerophosphate, 2 mM sodium pyrophosphate, 10% glycerol, 1 mM sodium orthovanadate, 1 mM phenylmethysulfonyl fluoride, and 1 mM benzamidine). Lysates were centrifuged at 20,000× *g* for 10 min to remove cellular debris. Whole-cell extracts were prepared and 50 μg of protein were resolved by SDS-PAGE and transferred to Immobilon-P membranes (Millipore, Bedford, MA; http://www.millipore.com) for detection with anti-p53 (#2524, Cell Signaling, Denver, MA; http://www.cellsignal.com), Caspase-3 (#9665, Cell Signaling), Caspase-9 (#9504, Cell Signaling), c-Myc (sc-764, Santa Cruz), Oct4 (sc-5279, Santa Cruz), Sox2 (sc-20088, Santa Cruz), Nanog (sc-30328, Santa Cruz), phosphor-Stat3 (#9131, Cell Signaling), Stat3 (sc-482, Santa Cruz), Lamin B (sc-6216, Santa Cruz) and β-actin (sc-47778, Santa Cruz) antibodies. The membranes were blocked with blocking solution (5% skim milk in TBS; 50 mM Tris-base, pH 7.4, 0.15 M NaCl, and 0.1% Tween-20) for 1 h, and incubated with primary antibodies in blocking solution for 16 h. The membranes were washed three times for 10 min in TBS and then incubated with HRP-conjugated anti-mouse or anti-rabbit antibodies (0.1 Ag/ml) for 1 h. Immunoreactivity was detected by enhanced chemiluminescence (ECL; Amersham, Piscataway, NJ; http://www.amersham biosciences.com).

### Immunofluorescence staining

MESCs were cultured on gelatin-coated cover slips. After washing twice with PBS, cells were fixed with 4% paraformaldehyde for 15 min. The cover slips were washed three times with PBS and the cells were permeabilized with 0.1% Tween-20 in PBS for 20 min followed by blocking for 30 min using blocking buffer (5% bovine serum albumin in PBS). After overnight incubation with the primary antibodies, the cover slips were washed three times with PBS and treated with Alexa Fluor 488 donkey anti-mouse IgG (Cat No: A21202, Invitrogen) or Alexa Flour 594 donkey anti-rabbit IgG (Cat No: A21207, Invitrogen) for 1 h in the dark. The cover slips were then washed three times in PBS and mounted with VECTASHIELD Mounting Medium with DAPI (Cat No: H-1200, Vector Laboratories, Burlingame, CA, USA; http://www.vectorlabs.com). Images were captured using an inverted microscopy system (ECLIPSE E600; Nikon, Kanagawa, Japan; http://www.nikon.com) and the analysis was performed using INFINITY2-1C software (Innerview 2.0, Lumenera, Canada; http://www.LUMENERA.com).

### Teratoma formation

For the teratoma formation assay, mESCs were trypsinized, and 1×10^6^ cells were suspended in PBS. The cell/PBS suspension was injected subcutaneously into Balb/c nude mice (Orient, Korea; http://www.orient.ac.kr). After teratoma formation, xenotransplantation masses were harvested, fixed in 10% phosphate-buffered formalin, and subsequently embedded in paraffin using a Tissue-Tek VIP embedding machine (Miles Scientific, Naperville, IL) and a Thermo Shandon Histocenter 2 (Thermo Fisher Scientific, Waltham, MA; http://www.thermofisher.com). Ten-micrometer sections were cut using a Leica RN2065 (Leica, Wetzlar, Germany; http://www.leica.com.), stained with hematoxylin and eosin (H&E), and analyzed by a trained pathologist. The experiments were reviewed and approved by the Institutional Animal Care and Use Committee of CHA University. All procedures were performed in accordance with the Guidelines for the Care and Use of Laboratory Animals published by the US National Institutes of Health (NIH publication no. 85-23, revised 1996).

### Statistical analysis

Graphical data are presented as the mean ± SD. Each experiment was performed at least three times and subjected to statistical analysis. Statistical significance between two groups was determined using the Student's *t*-test, and a *p* value<0.05 was considered significant. All statistical analyses were performed using the SAS statistical package, v.9.13 (SAS Inc., Cary, NC; http://www.sas.com/).

## Results

### Expression of *Ell3* in mouse ESCs

**Figure 1:**
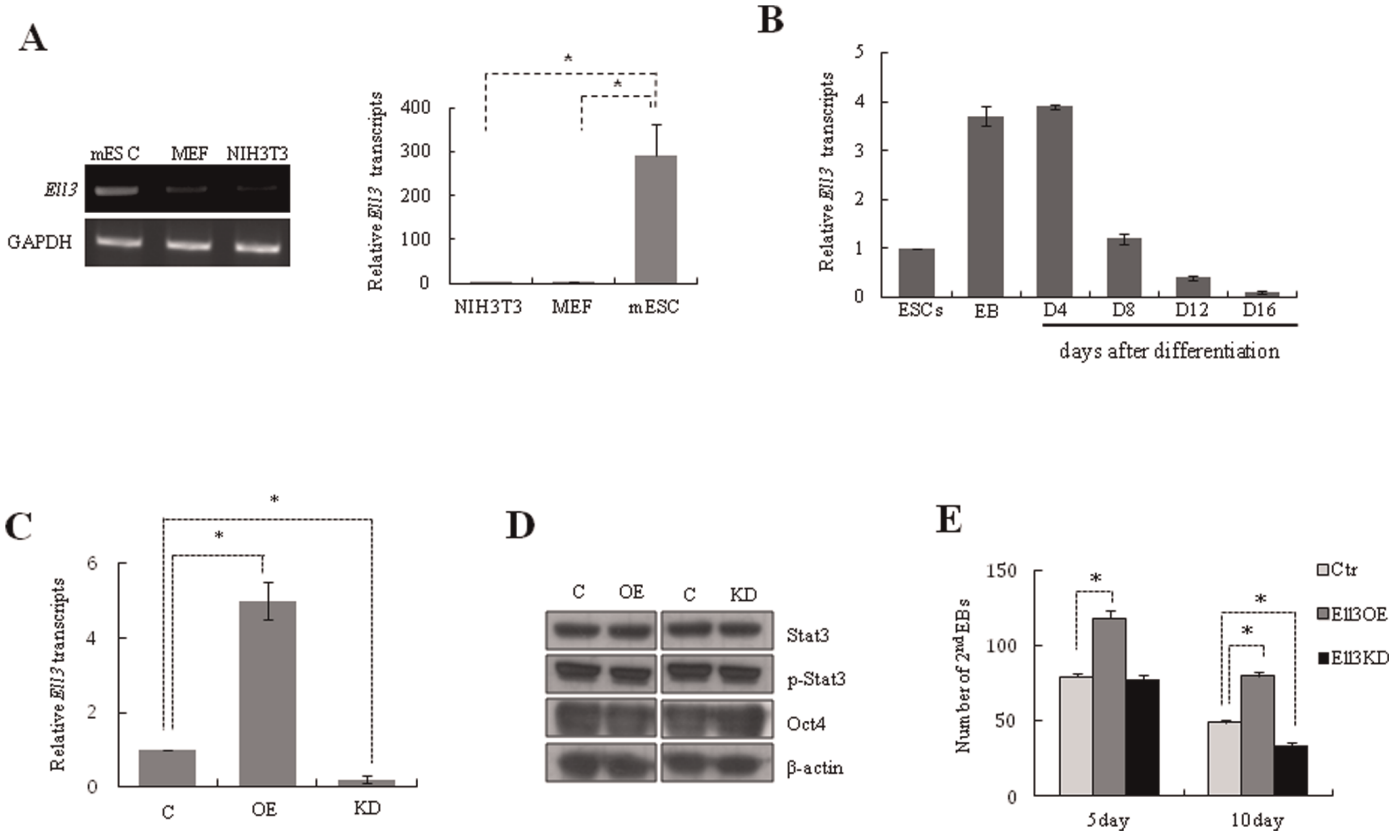
*Ell3* is specifically expressed in mESCs. (A) RT-PCR and real-time RT-PCR analysis shows that *Ell3* is expressed in mESCs but not in MEF and NIH3T3 cells. (B) Quantitative RT-PCR analysis of *Ell3* in ESCs, EBs, and differentiated cell stages (RA-D4, RA-D8, RA-D12 and RA-D16). *Ell3* transcripts increased at the EB and RA-D4 stages, and subsequently decreased as differentiation progressed (RA-D8, RA-D12 and RA-D16). (C) *Ell3* transcript levels in *Ell3-*OE and KD cells at passage 10 were compared with those in control cells. Passage was counted after *Ell3-*OE and KD stable cell lines were established. (D) Expression of Stat3, p-Stat3, and Oct4 in *Ell3*-OE and KD cells was compared with that in control mESCs. β-actin was used as a loading control for immunoblot analysis. (E) Primary EBs of *Ell3*-OE, *Ell3*-KD, and control mESCs were dissociated into single cells and re-seeded at a density of 1×10^6^ cells/mL in the same medium. The number of secondary EBs was counted under a bright microscope (n>3). All experiments were performed at least in triplicate, and all values represent the mean ± s.d. from at least triplicate experiments. *Indicates significant (P<0.05) results (Student's t-test).

In a previous study, we reported that comparing the gene expression profiles of oocytes and ESCs with those of differentiated cells is a valuable approach to identifying novel factors involved in the regulation of self-renewal or pluripotency of ESCs [13]. Comparison of the immature oocyte specific transcriptome, which was previously obtained using the annealing control primer-polymerase chain reaction (ACP-PCR) technique [22], with that of mESCs revealed that both oocytes and mESCs express *Ell3*, a testis-specific RNA polymerase II elongation factor. As shown in Fig. 1A, *Ell3* is actively expressed in mESCs, but the transcripts are weakly detected in differentiated cells such as mouse embryonic fibroblasts (MEFs) and NIH3T3 cells. These results suggest that *Ell3* may be confined to the undifferentiated state of mESCs. To test this idea, the expression level of *Ell3* was analyzed in mESCs treated with retinoic acid (RA) to induce differentiation. Surprisingly, expression of *Ell3* transiently increased during EB formation and in the early stages of spontaneous differentiation (up to 4 days), but subsequently decreased as differentiation progressed (Fig. 1B), suggesting that *Ell3* may play a role in the early differentiation of mESCs. To investigate the function of *Ell3* in mESCs, stable *Ell3-*OE or KD mES cell lines were generated. Analysis of *Ell3* mRNA in the OE or KD cell lines confirmed that the expression level of *Ell3* was stably maintained (Fig. 1C).

LIF and its cognate signaling pathway through Jak/Stat3 are crucial for self-renewal and pluripotency in mESCs [13], [14]. Phospho-Stat3 levels were therefore examined in *Ell3*-OE and KD cell lines. As shown in Fig. 1D, the phospho-Stat3 levels in *Ell3-*OE or KD cell lines were similar to those of control cells. In addition, the level of Oct4, a self-renewal marker of ESCs, was not affected by the change in *Ell3* expression. These results indicate that changes in *Ell3* levels do not affect the expression of major factors governing self-renewal of mESCs.

The efficiency of secondary EB formation, which reflects the capacity of ESCs to maintain an undifferentiated state and self-renewal capacity [23], was examined next. Surprisingly, the efficiency of secondary EB formation in *Ell3*-OE cells was 50% higher than that in control cells, while in *Ell3-*KD cells the secondary EB formation efficiency was lower than that in control cells when measured 10 days after EB formation (Fig. 1E). This result correlates with the finding that *Ell3* expression increases during EB formation (Fig. 1B) and strongly suggests that the expression level of *Ell3* affects the efficiency of EB formation, even though it does not regulate *Stat3* signaling or the expression of self-renewal markers.

### *Ell3* regulates pluripotent differentiation of mESCs

**Figure 2:**
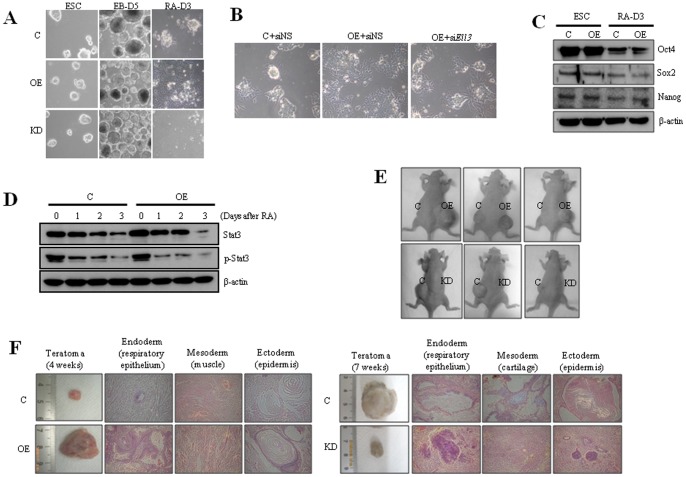
*Ell3* expression level in mESCs affects the differentiation capacity of the cells both in vitro and in vivo. (A) Five-day-old EBs (EB-D5) or differentiated *Ell3*-OE, *Ell3*-KD, and control mESCs were examined under a bright microscope. Differentiation was induced for 3 days (RA-D3) by removing LIF and adding retinoic acid (RA) to the mESCs culture media. (B) Nonspecific siRNA (siNS) or siRNAs targeting *Ell3* (si*Ell3*) were transfected into *Ell3*-OE cells in the ESCs state, and transfected cells were spontaneously differentiated for 2 days. Differentiating cells were examined under the microscope. (C) Expression of Oct4, Sox2, and Nanog in *Ell3*-OE cells was compared with that in control cells both in the ESC and differentiated states (RA-D3). β-actin was used as the loading control for immunoblot analysis. (D) Expression levels of Stat3 and phospho-Stat3 were compared between *Ell3-*OE and control cells as differentiation progressed. β-actin was used as the loading control for immunoblot analysis. (E) *Ell3-*OE, *Ell3*-KD, and control mESCs were injected into Balb/c nude mice and teratoma development was monitored. Teratomas of *Ell3*-OE and KD cell-injected mice were compared with those of control animals at 4 and 7 weeks after injection, respectively. (F) Morphology of mESCs teratomas obtained from Balb/c nude mice injected with either control, *Ell3*-OE, or *Ell3*-KD cells. MESC-induced teratomas were stained with H&E. Respiratory epithelium, muscle, cartilage, and epidermis were examined. All experiments were performed at least in triplicate.

The morphology of *Ell3*-OE and KD mESCs was indistinguishable from that of control cells under self-renewal or EB forming conditions (Fig. 2A). As in control cells, *Ell3* expression levels in *Ell3*-OE and KD cells increased as mESCs differentiated into EB or underwent RA-induced differentiation (Fig. S1). However, *Ell3*-OE cells differentiated more rapidly than control cells, while *Ell3*-KD cells were resistant to differentiation and showed a cell death phenotype when exposed to RA (Fig. 2A). The expression of lineage markers such as *nestin*, *gata4,* and *brachyury-T* was markedly increased in EBs or differentiated *Ell3*-OE cells compared to control cells, whereas the decrease of self-renewal marker expression was similar between *Ell3*-OE and control cells (Fig. S2). We then examined whether suppression of *Ell3* expression could inhibit the enhanced differentiating capacity of *Ell3*-OE cells. Indeed, transfection of siRNA targeting *Ell3* attenuated the enhanced differentiation capacity of *Ell3*-OE cells (Fig. 2B).

Despite the enhanced differentiation potential of *Ell3*-OE cells, the expression of self-renewal factors such as Oct4, Sox2 and Nanog during differentiation decreased in similar levels in control and *Ell3*-OE cells (Fig. 2C). In addition, Stat3 and p-Stat3 were also decreased to a similar extent in the differentiating control and *Ell3*-OE cells (Fig. 2D). These results suggest that the enhanced differentiation of *Ell3*-OE cells is not associated with alterations in the self-renewal capacity of mESCs.

To confirm that *Ell3* plays a role in pluripotent differentiation *in vivo*, *Ell3*-OE or KD mESCs were injected into Balb/c nude mice, and teratoma formation was monitored. As shown in Fig. 2E, teratoma development occurred more rapidly in *Ell3*-OE cell-injected animals than in controls, while *Ell3*-KD cell-injected mice did not develop teratomas until 7 weeks following transplantation. When examined by histological staining, teratomas harvested 4 weeks after transplantation of *Ell3*-OE cells showed well developed differentiated tissues consisting of all three germ layers: respiratory epithelium (endoderm), muscle (mesoderm), and epidermis (ectoderm) (Fig. 2F, left panel). By contrast, teratomas from *Ell3*-KD cells did not show the typical staining of specific lineage cell types (Fig. 2F, right panel). Taken together, these results indicate that *Ell3* regulates the pluripotent differentiation of mESCs.

### The effect of *Ell3* expression on the neural differentiation of mESCs

**Figure 3:**
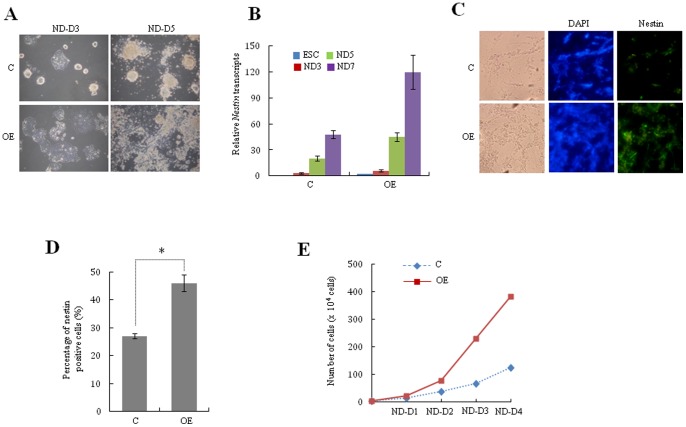
*Ell3* expression promotes neural differentiation of mESCs. (A) *Ell3*-OE and control mESCs were differentiated into neural lineage, and differentiating cells were examined under a bright microscope 3 days (ND-D3) and 5 days (ND-D5) after differentiation. (B) Nestin expression in *Ell3*-OE cells was compared with that in control cells by qRT-PCR 0, 3, 5, and 7 days after neural differentiation (ND0, ND3, ND5, and ND7, respectively). (C) Nestin expression in *Ell3*-OE cells was compared with that of control cells by immunostaining 7 days after neural differentiation. (D) Nestin^+^ cells in differentiating *Ell3*-OE and control cells were analyzed by FACS. MESCs were cultured in neural differentiation media for 7 days and 1×10^4^ cells were analyzed for nestin expression by FACS. (E) The number of adherent cells was counted at the indicated days after 1×10^4^ cells of *Ell3*-OE and control mESCs were differentiated into the neural lineage. All experiments were performed at least in triplicate and all values represent the mean ± s.d. from at least triplicate experiments. * Indicates significant (P<0.05) results (Student's t-test).

To further investigate the effect of *Ell3* on lineage specific differentiation, we compared the neural differentiation of *Ell3*-OE cells to that of control cells. Compared with control cells, *Ell3*-OE cells rapidly lost ESC morphology within 3 days in neural induction media and showed significantly enhanced differentiated morphology during differentiation (Fig. 3A). *Ell3* expression increased during neural differentiation both in control and *Ell3*-OE cells (Fig. S3). Quantitative analysis of nestin mRNA supported the notion that *Ell3*-OE cells differentiated into neural cells more rapidly than control cells (Fig. 3B). Immunocytochemical staining showed that nestin-expressing cells were abundant among the differentiating *Ell3*-OE cells than among the control cells (Fig. 3C). This result was further confirmed by flow cytometry analysis of nestin^+^ cells sorted using a fluorescence activated cell sorting (FACS) assay. *Ell3*-OE cells contained a significantly higher proportion of nestin^+^ cells than control cells when 1×10^4^ differentiating cells were analyzed by FACS, indicating that *Ell3* expression promotes the neural differentiation of mESCs (Fig. 3D). Furthermore, we compared the number of differentiating cells between *Ell3*-OE and control cells. When 1×10^4^ mESCs were cultured in neural differentiating medium for 4 days, the number of *Ell3-*OE cells was approximately 4 times higher than that of control cells (Fig. 3E). Taken together, these data suggest that *Ell3* facilitates the proliferation of differentiating cells and promotes the neural differentiation of mESCs.

### Activation of *Zeb1* expression is the major cause of enhanced differentiation of *Ell3*-overexpressing mESCs

**Figure 4:**
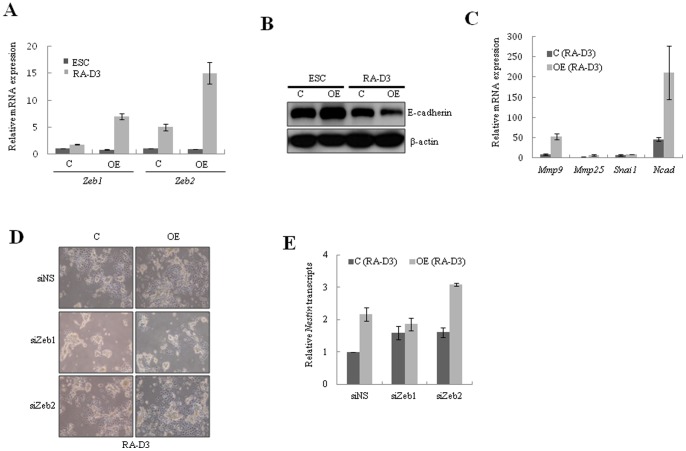
Suppression of *Zeb1* attenuates the enhanced differentiation of *Ell3*-OE cells. (A) The expression of *Zeb1* and *Zeb2* in the ESC state or RA-induced differentiating state of control or *Ell3*-OE cells was analyzed by real-time RT-PCR. (B) The expression of E-cadherin in the ESC state or RA-induced differentiating state of ESC or *Ell3*-OE cells was analyzed by immunoblot analysis. β-actin was used as a loading control. (C) The expression of EMT markers during RA-induced differentiation of mESCs was compared between control and *Ell3*-OE cells by real-time RT-PCR. (D) Control or *Ell3*-OE cells were transfected with siRNAs targeting *Zeb1* or *Zeb2* and spontaneously differentiated 1 day after transfection. Cell morphology was examined 3 days after RA-induced differentiation (RA-D3). Nonspecific siRNA was transfected as a control. (E) The expression of *Nestin* was analyzed by real-time RT-PCR 3 days after RA-induced differentiation of si*Zeb1-* or si*Zeb2-*transfected control or *Ell3*-OE cells. All experiments were performed at least in triplicate and all values represent the mean ± s.d. from at least triplicate experiments. * Indicates significant (P<0.05) and ** highly significant (p<0.01) results (Student's t-test).

Recently, it was shown that EMT is an early and essential step in lineage specification of ESCs [9]. To investigate the underlying mechanism of *Ell3* promotion of mESC differentiation, we compared the gene expression patterns of *Zeb1* and *Zeb2*, core transcription factors that induce EMT, between *Ell3*-OE and control cells. As expected, the expression of *Zeb1* and *Zeb2* was significantly higher in *Ell3*-OE cells than in control cells, both in self-renewal and differentiating states (Fig. 4A). Since Zeb factors are core transcriptional repressors that suppress the expression of epithelial genes, including *E-cadherin*, as well as stemness-inhibiting microRNAs [24], [25], we analyzed the expression of E-cadherin to examine whether EMT was increased during the differentiation of *Ell3*-OE cells. As expected, immunoblot analysis showed that E-cadherin expression in differentiating *Ell3-*OE cells was lower than that in control cells (Fig. 4B). In contrast to *E-cadherin*, *N-cadherin* induces invasion, migration, and EMT of multiple cancer cell lines [26], [27]. Consistently, N-cadherin and other EMT markers such as Mmp9 and Mmp25 were significantly expressed during the differentiation of *Ell3-*OE cells (Fig. 4C). These results suggest that the enhanced differentiation capacity of *Ell3*-OE cells may be due to the rapid induction of EMT.

Next, we analyzed whether *Zeb1* and *Zeb2* are downstream targets of *Ell3* in mESCs. MESCs were transfected with siRNAs targeting *Zeb1* or *Zeb2* and examined for phenotypic changes. Interestingly, the enhanced differentiation capacity of *Ell3*-OE cells was compromised by the transfection of *Zeb1* siRNA, whereas suppression of *Zeb2* did not affect the differentiation of mESCs (Fig. 4D). Consistently, the expression of *Nestin*, which was enhanced during the differentiation of *Ell3-*OE cells (Fig. 3B), was also suppressed by the knockdown of *Zeb1* (Fig. 4E). However, knockdown of *Zeb2* in *Ell3*-OE cells did not suppress the enhanced expression of *Nestin*. Taken together, these results suggest that *Zeb1*, but not *Zeb2*, is a downstream target of *Ell3* that induces EMT during the differentiation of mESCs.

### Apoptosis of differentiating *Ell3* KD is associated with enhanced p53 expression and caspase pathway activation

**Figure 5:**
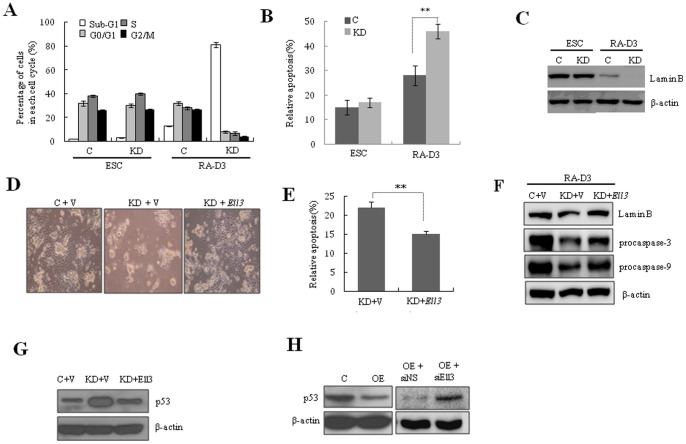
Differentiating *Ell3*-KD cells undergo apoptosis, which is associated with enhanced p53 expression and activated caspase pathway. (A) Cell cycle distribution of control and *Ell3*-KD cells stained with propidium iodide (PI). Cells in the ESC state or differentiated for 3 days (RA-D3) by removing LIF and adding retinoic acid (RA) were analyzed by flow cytometry. (B) Apoptosis of control and *Ell3-*KD cells was quantitatively analyzed either in the ESC or differentiated state by determining the number of Annexin V-positive cells. Cells were spontaneously differentiated for 3 days (RA-D3). (C) The amounts of Lamin B in control or *Ell3*-KD cells were determined either in the ESC state or in spontaneously differentiated cells for 3 days (RA-D3) by immunoblot analysis. β-actin was used as a loading control. Control (V) or *Ell3*-expressing vectors (*Ell3*) were transfected into *Ell3*-KD cells in the ESC state, and transfected cells were spontaneously differentiated for 3 days. Cells were examined under the microscope (D) and apoptosis was quantitatively analyzed by determining the number of Annexin V-positive cells (E). (F) The amounts of Lamin B, procaspase-3, and procaspase-9 in *Ell3*-KD cells transfected with control or *Ell3*-expressing plasmids were determined either in the ESC state or in spontaneously differentiated cells after 3 days by immunoblot analysis. β-actin was used as a loading control. (G) *Ell3*-KD cells were transfected with control (V) or *Ell3*-expressing plasmids (Ell3). Transfected cells were spontaneously differentiated for 3 days, and *p53* levels were examined by immunoblot analysis. β-actin was used as a loading control. (H) *p53* in control or *Ell3*-OE cells was determined after 3 days of spontaneous differentiation by immunoblot analysis (left panel). *Ell3*-OE cells were transfected with nonspecific siRNA (siNS) or *Ell3*-targeting siRNA (si*Ell3*). Transfected cells were spontaneously differentiated for 3 days, and *p53* was examined by immunoblot analysis (right panel). β-actin was used as a loading control. All experiments were performed at least in triplicate and all values represent the mean ± s.d. from at least triplicate experiments. * Indicates significant (P<0.05) and ** highly significant (p<0.01) results (Student's t-test).

As shown in Fig. 2A, differentiating *Ell3*-KD cells showed a cell death phenotype. When we analyzed the cell cycle, we found that the sub-G1 population in differentiating *Ell3*-KD cells was significantly increased compared with that of the control cells (Fig. 5A). In addition, Annexin V/PI staining showed increased cell death in differentiating *Ell3*-KD cells compared with control cells (Fig. 5B). As caspases are key molecules in apoptosis, the possible relationship between caspase activation and cell death of *Ell3*-KD cells was estimated by analyzing the amount of Lamin B, a proteolysis substrate for activated caspase-3 and -6 [28], [29]. Immunoblotting results showed complete degradation of Lamin B protein in differentiating *Ell3*-KD cells, confirming increased activity of the caspase pathway in *Ell3*-KD cells compared with control cells during differentiation (Fig. 5C). To confirm that apoptosis in differentiating *Ell3*-KD was indeed caused by *Ell3* suppression, we ectopically transfected an *Ell3-*expressing plasmid into *Ell3*-KD cells and induced differentiation. Expectedly, introduction of an *Ell3*-expressing plasmid into *Ell3*-KD cells prevented apoptotic cell death and induced differentiation comparable to that of control cells in differentiation media (Fig. 5D). Apoptosis of *Ell3*-KD cells under differentiation conditions significantly decreased with the re-expression of *Ell3* (Figure 5E). To confirm that activation of the caspase pathway during the differentiation of mESCs depends on *Ell3* expression, the amounts of Lamin B, procaspase-3, and procaspase-9 were analyzed after the forced expression of *Ell3* in *Ell3*-KD cells. As shown in Fig. 5F, *Ell3*-KD cells transfected with an *Ell3*-expressing plasmid expressed higher amounts of Lamin B, procaspase-3, and procaspase-9 3 days after RA-induced differentiation, which indicates that forced expression of *Ell3* in *Ell3*-KD inhibited activation of the caspase pathway in differentiated mESCs. These results indicate that *Ell3* regulates caspase-dependent apoptosis in mESCs during differentiation.

Since the C-terminal domain of Ell3 shows strong similarities to that of Ell, which acts as a negative regulator of p53 [20], [21] (a major mediator of apoptosis in mammalian cells), we investigated whether *Ell3* expression affects the amount of p53 in differentiating mESCs. Indeed, the p53 protein level, which was significantly higher in differentiating *Ell3*-KD cells compared with control cells, returned to the control cell level when *Ell3*-KD cells were transfected with an *Ell3*-expressing plasmid (Fig. 5G). These results suggest that *Ell3* functions as a negative regulator of p53 in differentiating mESCs. Consistently, p53 expression in differentiating *Ell3*-OE cells was significantly lower than that in control cells, and depletion of *Ell3* by siRNA resulted in an increase in p53 protein levels (Fig. 5H, Fig. S4A). Furthermore, *Ell3* siRNA-mediated depletion of *Ell3* enhanced apoptosis in *Ell3*-OE cells during differentiation (Fig. S4B). These results show that changes in p53 protein expression in differentiating *Ell3*-OE or KD mESCs depend on the expression level of *Ell3.*

## Discussion

Our data establish a model whereby *Ell3* promotes EMT and suppresses *p53* levels, which leads to the initiation of differentiation of mESCs. We showed that *Ell3* overexpression promotes differentiation of mESCs with the concomitant activation of EMT marker genes. In addition, *Ell3*-knock-down mESCs undergo apoptosis along with an accumulation of *p53*. Interestingly, we found that the activation of *Zeb1*, which is known to link EMT-activation and stemness maintenance in mESCs [24], is an essential event for *Ell3* to promote differentiation of mESCs, as shown by the finding that suppression of *Zeb1* in *Ell3*-OE cells compromises the differentiation-promoting effect of *Ell3*. Based on these results, we propose that the promotion of EMT may account for the role of *Ell3* in ESC differentiation, which is in line with a previous study demonstrating that EMT is an early and essential step in the differentiation of ESCs [9]. Since *Ell3* is known as a transcription elongation factor, it would be important to elucidate whether *Ell3* directly regulates the expression of *Zeb1* and *Zeb2*. The mechanism by which *Ell3* activates EMT marker genes, including *Zeb1 and Zeb2,* is currently under investigation in our laboratory.

Another important advance of this study is the discovery that *p53* protein stability is enhanced, and the caspase pathway is activated, when *Ell3* expression is suppressed in differentiating mESCs. *P53* functions as a decision maker in mESCs, inducing differentiation by repressing *Nanog* expression, [30] or inhibiting differentiation by inducing expression of several WNT ligands [31]. *In vitro* differentiation of mESCs results in decreased levels of *p53* and shifts p53 conformational status to the mutant form, allowing differentiating cells to evade apoptosis [32]. Our study indicates that *Ell3* may function to safeguard differentiating ES cells by escaping apoptosis via suppression of p53.

*Ell3* overexpression or suppression did not affect the protein or transcript levels of *p53* in the self-renewal state of mESCs, indicating that *Ell3*-mediated regulation of *p53* was active only under differentiation conditions (Fig. S5). One possible hypothesis is that other factors induced during mESC differentiation may cooperate with *Ell3* to activate the *p53* degradation pathway when mESCs transition from self-renewal to differentiation. Additional studies are needed to elucidate how *Ell3* only affects *p53* expression in differentiating mESCs, but not in self-renewing mESCs.

Activation of caspase-3 induces differentiation of ESCs by inducing the cleavage of Nanog [33]. However, the enhanced activity of caspase-3 in *Ell3*-KD cells induced apoptosis instead of promoting differentiation. This result indicates that a more complex mechanism may underlie the involvement of caspase activity in the differentiation of ESCs. One possibility is that caspase levels may be regulated to balance the rates of differentiation and apoptosis in ESCs during differentiation.

The regulatory mechanism underlying how *Ell3* regulates *p53* expression remains elusive. *Ell3* did not have an affect on p53 transcription, suggesting that *Ell3* controlled *p53* levels by modulating *p53* protein stability (data not shown). Since Mdm2-mediated ubiquitination-dependent degradation is one of the main pathways negatively regulating p53 levels, future studies should examine whether *Ell3* regulates the level of p53 by the Mdm2-mediated ubiquitination pathway.

It remains unclear whether there is a link between the regulation of EMT and that of *p53* expression by *Ell3*. Indeed, *p53* loss of function or mutations was recently found to promote cancer cell EMT by de-repressing Snail 1 protein expression and activity [34]. Therefore, it would be interesting to study the link between p53 and EMT in the initiation of ESC differentiation.

## Supporting Information

Figure S1 ***Ell3*** **transcripts in** ***Ell3*** **-OE and KD cells during EB formation or RA-induced differentiation were quantitatively compared with those in control cells.** Five-day-old EBs (EB-D5) or cells differentiated for 3 days (RA-D3) were used for the analysis.
(TIF)

Figure S2 **(A)** ***Oct4, Sox2,*** **and** ***Nanog*** **expression in** ***Ell3*** **-OE and control mESCs was analyzed by real-time RT-PCR 0, 3, 5, and 7** **days after RA-induced spontaneous differentiation.** (B) Nestin, Gata4, and Brachyury-T expression in Ell3-OE and control mESCs was analyzed by real-time RT-PCR in 5 days old EBs (EB-D5) or in RA-induced differentiated cells (RA-D3).
(TIF)

Figure S3 **Expression level of** ***Ell3*** **during the neural differentiation of** ***Ell3*** **-OE or control mESCs was analyzed by real-time RT-PCR.**
(TIF)

Figure S4 ***Ell3*** **-OE cells were transfected with nonspecific siRNA (siNS) or** ***Ell3*** **-targeting siRNA (si** ***Ell3*** **) for 48** **h.** *Ell3* transcript levels were compared with those in control cells transfected with siNS (A), and apoptosis was quantitatively analyzed by determining the number of Annexin V-positive cells (B). All values represent the mean ± s.d. from at least triplicate experiments. ** Indicates highly significant (P<0.01) results (Student's t-test).
(TIF)

Figure S5 **RNA or protein levels of** ***p53*** **in** ***Ell3*** **-OE or control mESCs were analyzed by real-time RT-PCR or immunoblot analysis.**
(TIF)

Table S1 **Real time PCR primer sequences used in this study.**
(TIF)
