# Supplementary figures and images for: Ell3 Enhances Differentiation of Mouse Embryonic Stem Cells by Regulating Epithelial-Mesenchymal Transition and Apoptosis
Source: PLoS One. 2012 Jun 29;7(6):e40293. doi: 10.1371/journal.pone.0040293 (PMC3386972; doi:10.1371/journal.pone.0040293)

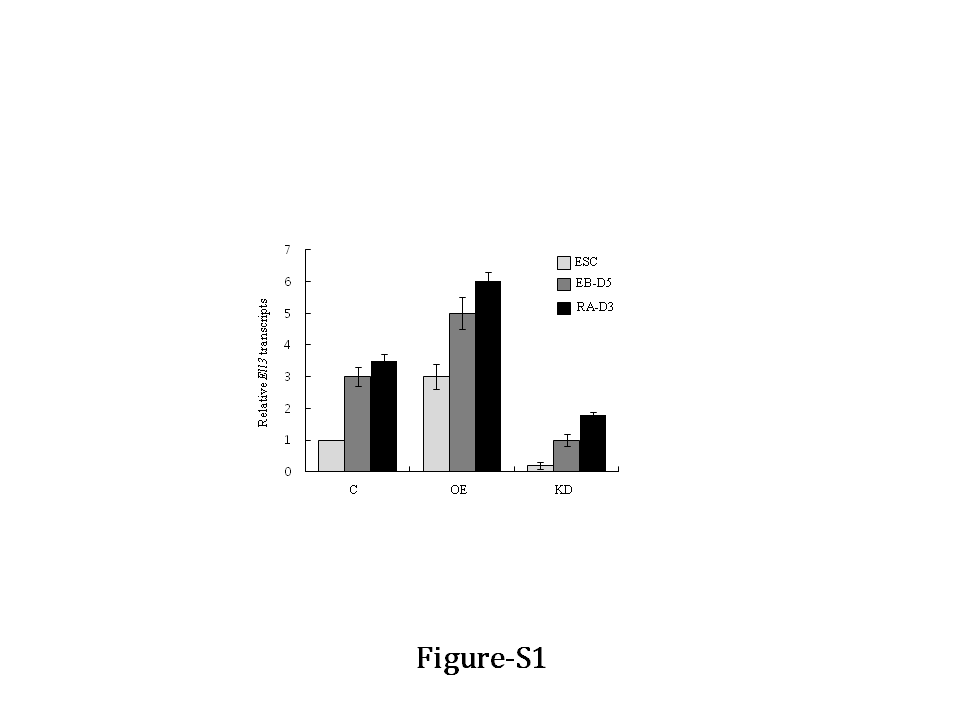

Supplement: Figure S1 — Ell3 transcripts in Ell3 -OE and KD cells during EB formation or RA-induced differentiation were quantitatively compared with those in control cells. Five-day-old EBs (EB-D5) or cells differentiated for 3 days (RA-D3) were used for the analysis. (TIF) [file pone.0040293.s001.tif]

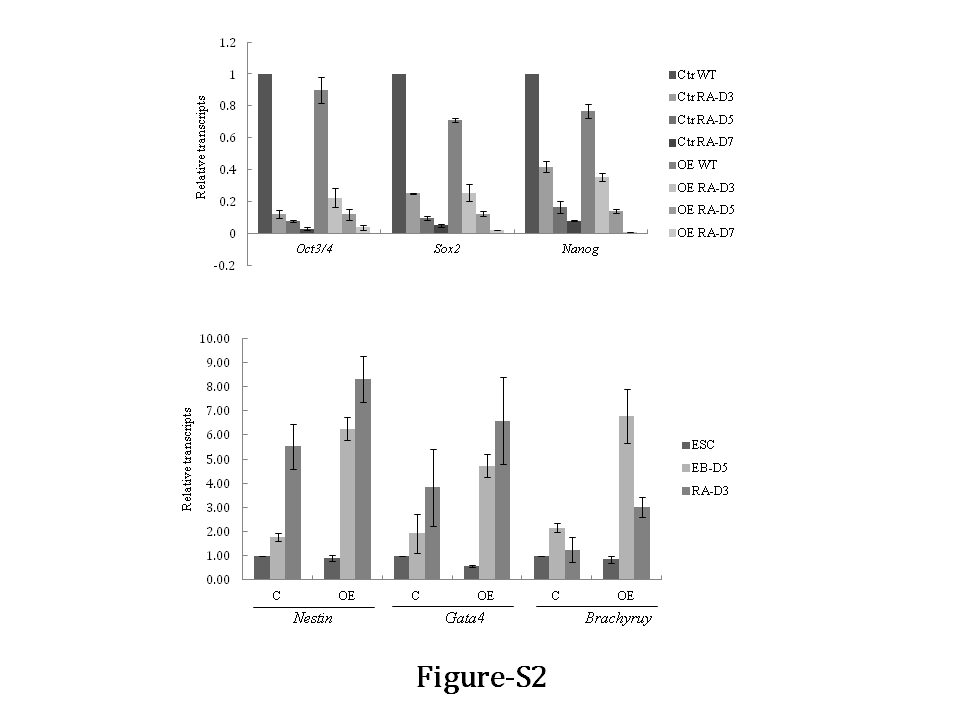

Supplement: Figure S2 — (A) Oct4, Sox2, and Nanog expression in Ell3 -OE and control mESCs was analyzed by real-time RT-PCR 0, 3, 5, and 7 days after RA-induced spontaneous differentiation. (B) Nestin, Gata4, and Brachyury-T expression in Ell3-OE and control mESCs was analyzed by real-time RT-PCR in 5 days old EBs (EB-D5) or in RA-induced differentiated cells (RA-D3). (TIF) [file pone.0040293.s002.tif]

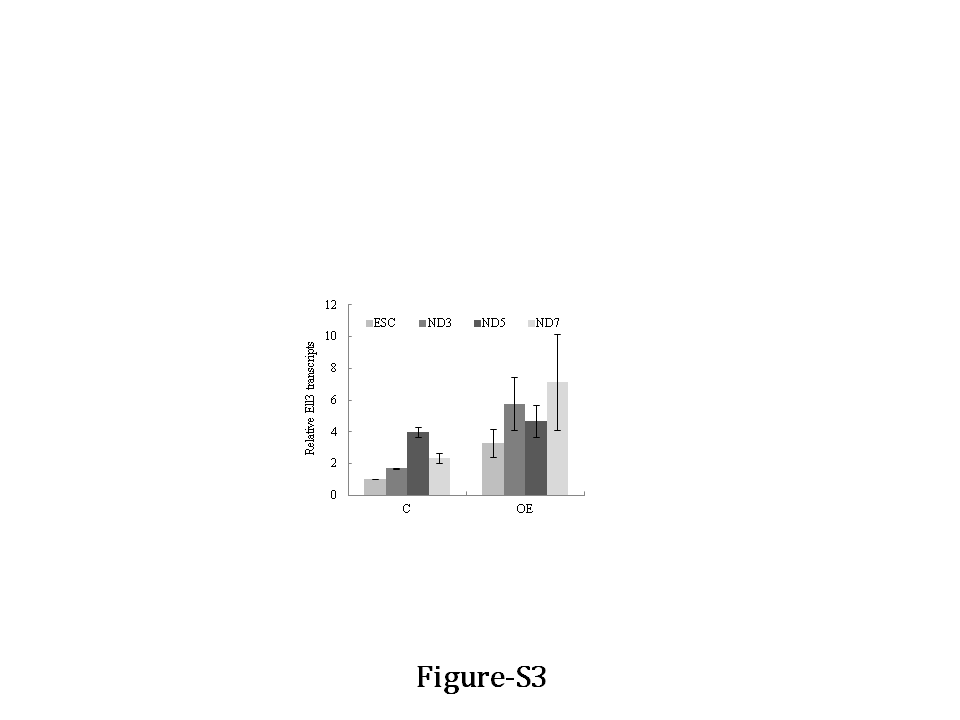

Supplement: Figure S3 — Expression level of Ell3 during the neural differentiation of Ell3 -OE or control mESCs was analyzed by real-time RT-PCR. (TIF) [file pone.0040293.s003.tif]

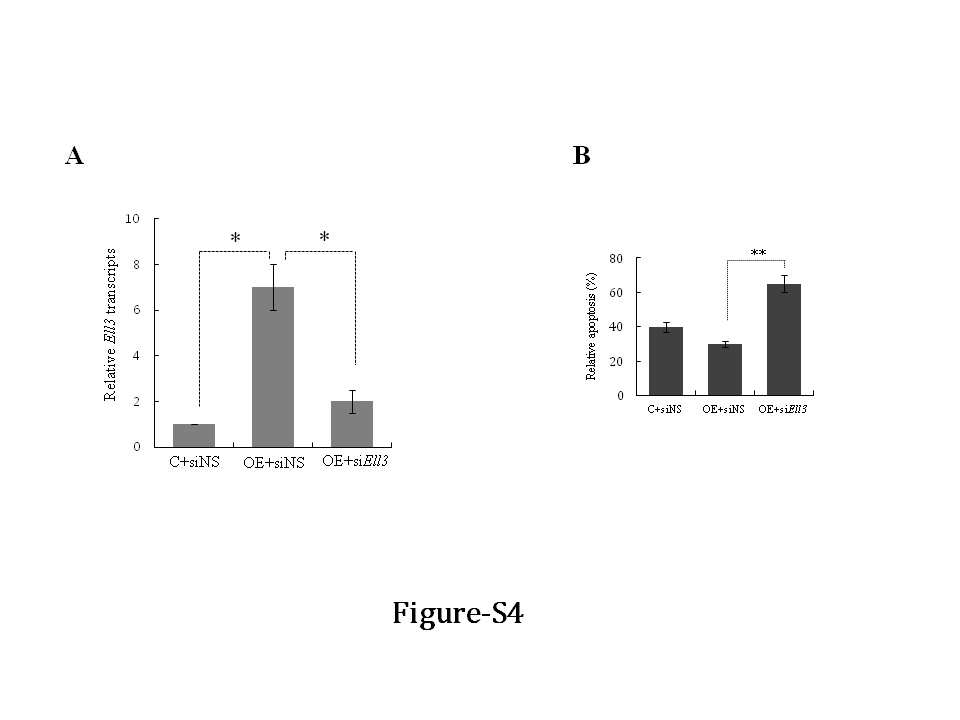

Supplement: Figure S4 — Ell3 -OE cells were transfected with nonspecific siRNA (siNS) or Ell3 -targeting siRNA (si Ell3 ) for 48 h. Ell3 transcript levels were compared with those in control cells transfected with siNS (A), and apoptosis was quantitatively analyzed by determining the number of Annexin V-positive cells (B). All values represent the mean ± s.d. from at least triplicate experiments. ** Indicates highly significant (P<0.01) results (Student's t-test). (TIF) [file pone.0040293.s004.tif]

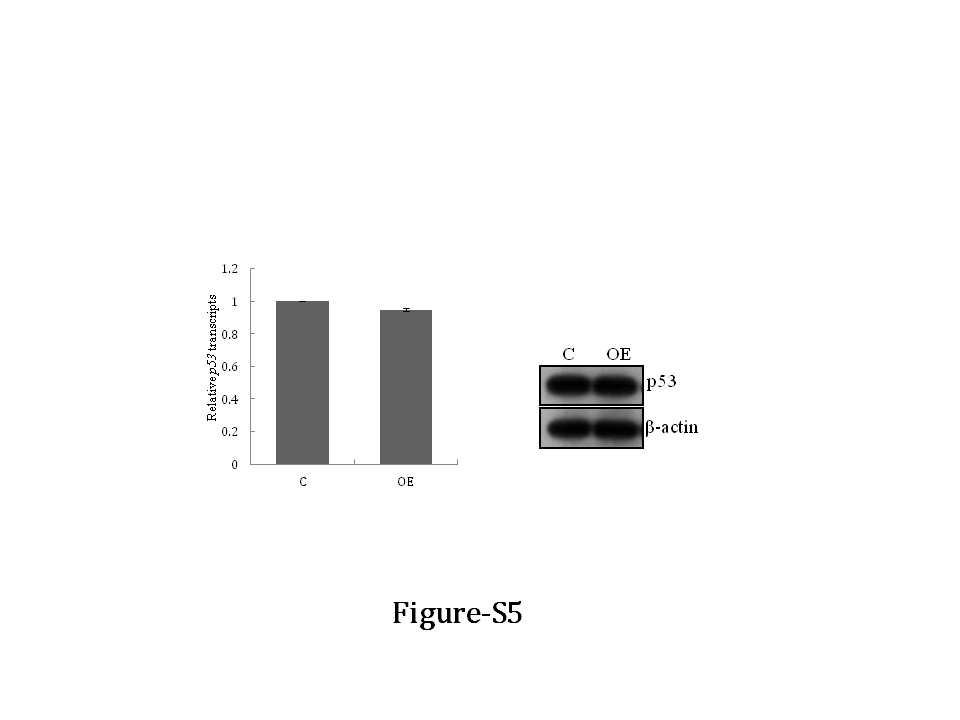

Supplement: Figure S5 — RNA or protein levels of p53 in Ell3 -OE or control mESCs were analyzed by real-time RT-PCR or immunoblot analysis. (TIF) [file pone.0040293.s005.tif]

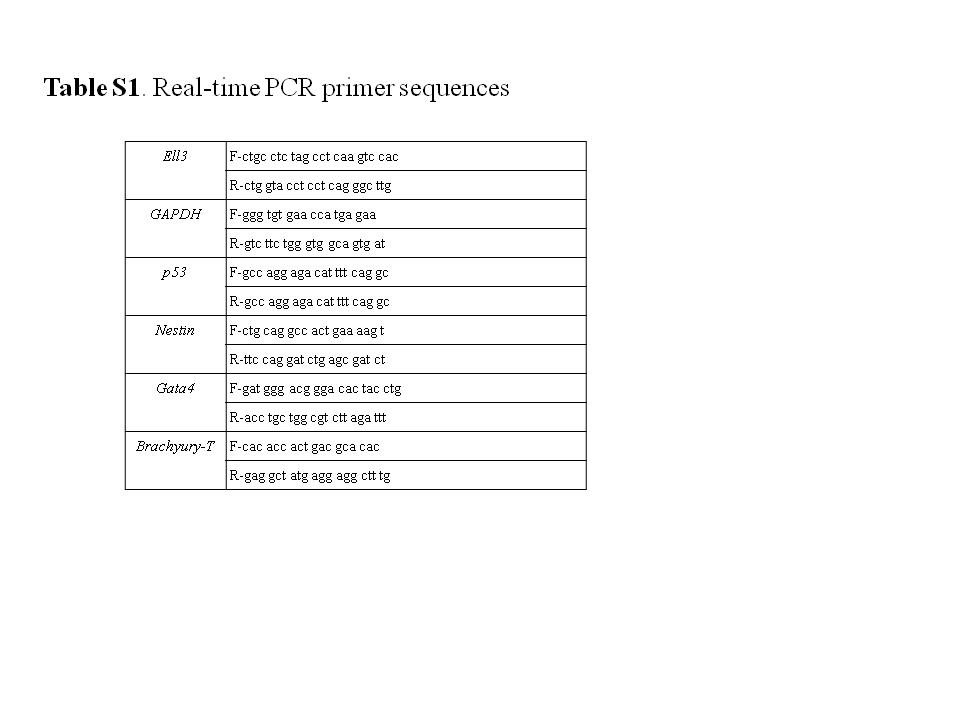

Supplement: Table S1 — Real time PCR primer sequences used in this study. (TIF) [file pone.0040293.s006.tif]
